# Supplementary material for: The genomic origin of the unique chaetognath body plan
Source: Nature. 2025 Aug 13;645(8082):964–73. doi: 10.1038/s41586-025-09403-2 (PMC12460157; doi:10.1038/s41586-025-09403-2)
Supplement: Supplementary file 1 — Supplementary Fig. 1–3 [file 41586_2025_9403_MOESM1_ESM.pdf]

nature portfolio

https://doi.org/10.1038/s41586-025-09403-2

Supplementary information

# The genomic origin of the unique chaetognath body plan

In the format provided by the authors and unedited

# Supplementary figures

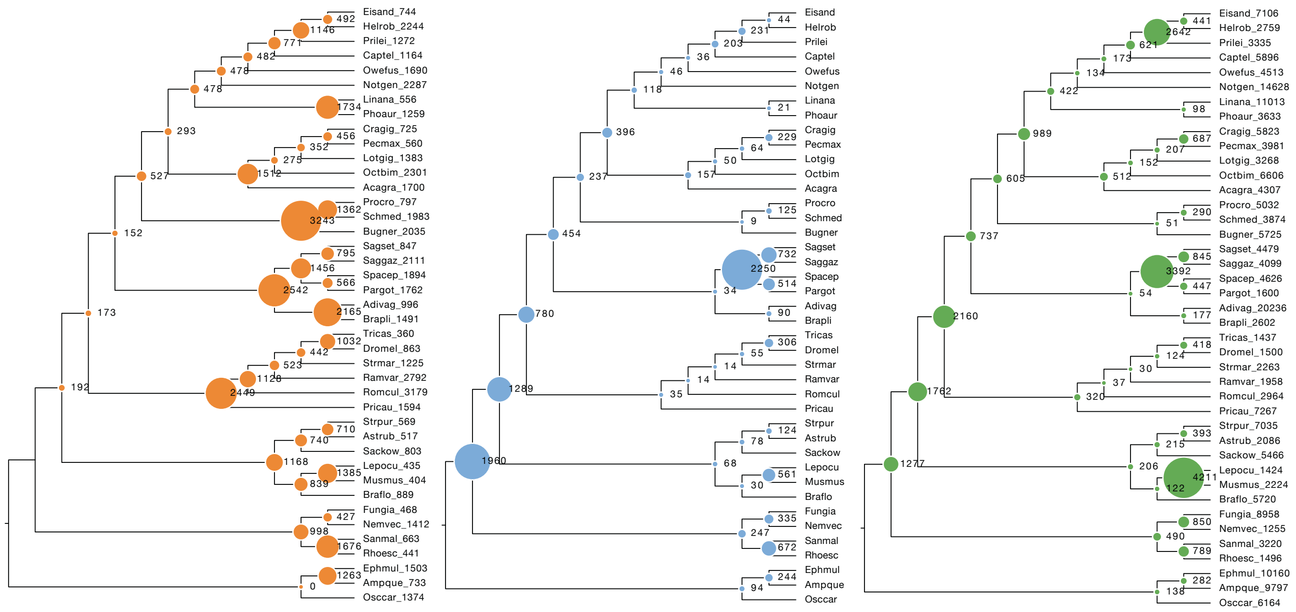


**Supplementary Figure 1.** Gene family events reconstructed using an alternative ('Rouphozoa') topology for spiralians, where platyhelminths are sister-group to bryozoans and constitute the first lineage of lophotrochozoans in order to assess the impact of the topology on  inferred gene gain and duplication events. From left to right, gene family loss (orange), gene family gain (blue) and gene duplication events (green).

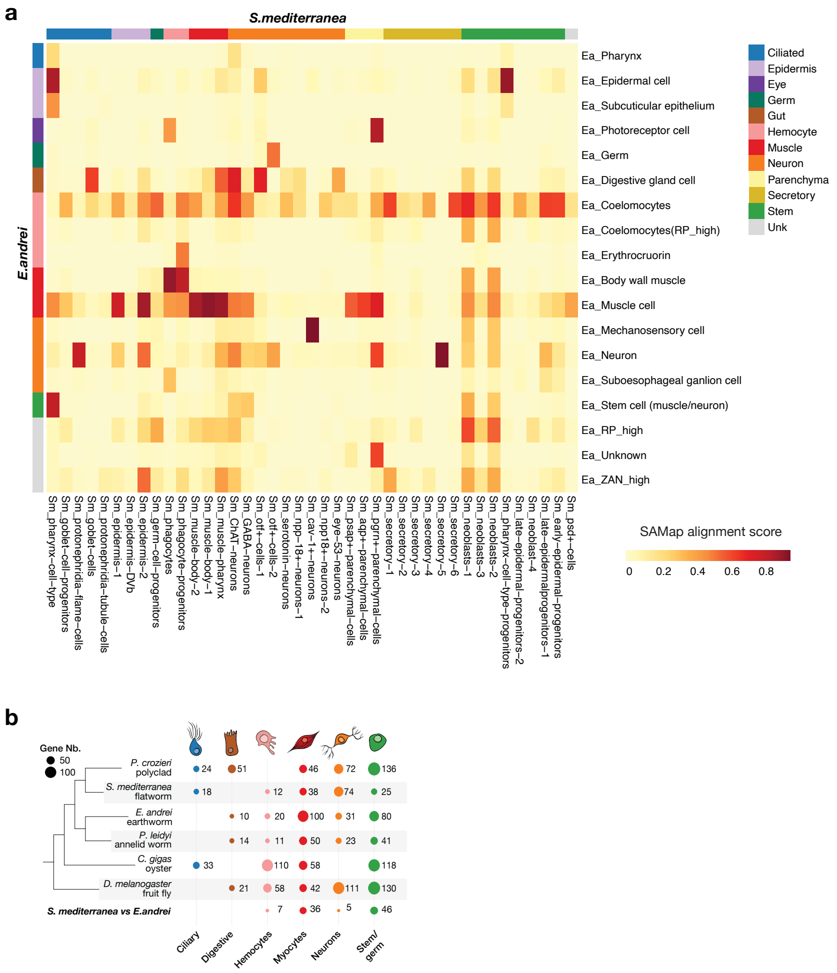


**Supplementary Figure 2.** Pairwise SAMap comparison between two lophotrochozoans: *Schmidtea mediterranea* and the annelid *Eisenia andrei.* **a,** Matrix of alignment scores across cell types and **b,** summary of comparison highlighting the number of genes found significantly co-expressed between cell types.

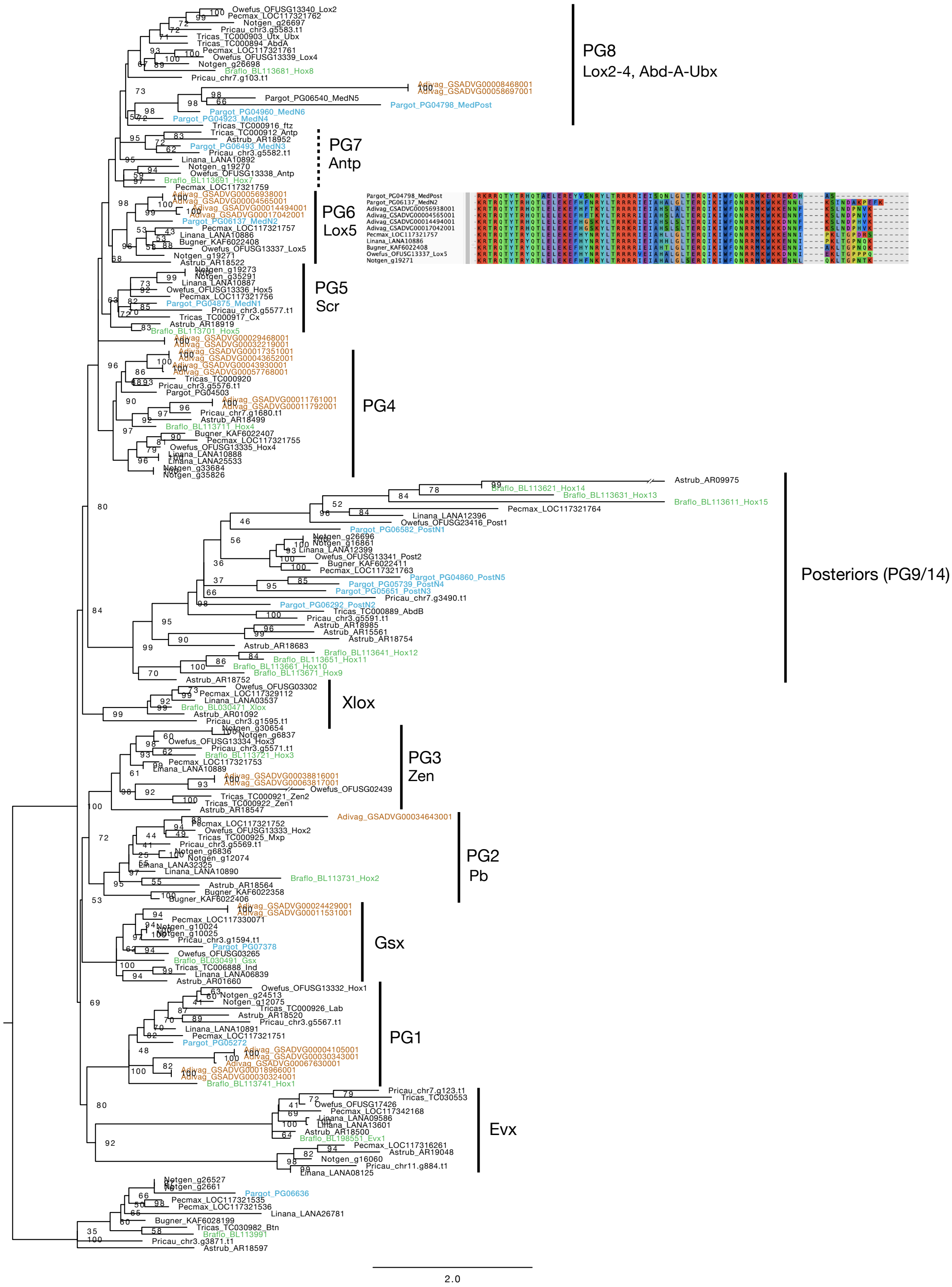


**Supplementary Figure 3. Phylogenetic assignment of chaetognath Hox genes.** Tree reconstruction using IQTREE assuming a LG+X model and supports derived from 1000 Ultrafast bootstrap. MedN1 is related to Hox5, MedN2 to Lox5, which is further confirmed by the presence of a divergent but identifiable Lox5 peptide (see excerpt), MedN3 is less clearly positioned, but the genes MedN4,5,6 are clustered in the tree and thus appear to represent lineage specific duplicates. Interestingly, our tree suggests a possible affinity of Lox2/Lox4 to these genes (with mild support), which could indicate that these median genes were independently expanded in chaetognaths (MedN4,5,6), spiralians (Lox2/4) and even ecdysozoans (Ubx/Abd-A). MedPost of chaetognath and rotifers cluster together with a supported association to MedN5,6 that could be explained by accelerated evoluton **b,** Presence of the Lox5 spiralian-specific peptide.
