# Supplementary material for: The genomic origin of the unique chaetognath body plan
Source: Nature. 2025 Aug 13;645(8082):964–73. doi: 10.1038/s41586-025-09403-2 (PMC12460157; doi:10.1038/s41586-025-09403-2)
Supplement: Supplementary file 2 — Reporting Summary [file 41586_2025_9403_MOESM2_ESM.pdf]

nature portfolio

Corresponding author(s): F. Marlétaez

Last updated by author(s): Jul 2, 2025

Reporting Summary

Nature Portfolio wishes to improve the reproducibility of the work that we publish. This form provides structure for consistency and transparency in reporting. For further information on Nature Portfolio policies, see our Editorial Policies and the Editorial Policy Checklist.

Statistics

For all statistical analyses, confirm that the following items are present in the figure legend, table legend, main text, or Methods section.

n/a	Confirmed	
☒	☐	The exact sample size (*n*) for each experimental group/condition, given as a discrete number and unit of measurement
☒	☐	A statement on whether measurements were taken from distinct samples or whether the same sample was measured repeatedly
☐	☒	The statistical test(s) used AND whether they are one- or two-sided<br>*Only common tests should be described solely by name; describe more complex techniques in the Methods section.*
☒	☐	A description of all covariates tested
☐	☒	A description of any assumptions or corrections, such as tests of normality and adjustment for multiple comparisons
☐	☒	A full description of the statistical parameters including central tendency (e.g. means) or other basic estimates (e.g. regression coefficient) AND variation (e.g. standard deviation) or associated estimates of uncertainty (e.g. confidence intervals)
☐	☒	For null hypothesis testing, the test statistic (e.g. *F*, *t*, *r*) with confidence intervals, effect sizes, degrees of freedom and *P* value noted<br>*Give P values as exact values whenever suitable.*
☒	☐	For Bayesian analysis, information on the choice of priors and Markov chain Monte Carlo settings
☒	☐	For hierarchical and complex designs, identification of the appropriate level for tests and full reporting of outcomes
☒	☐	Estimates of effect sizes (e.g. Cohen's *d*, Pearson's *r*), indicating how they were calculated

Our web collection on statistics for biologists contains articles on many of the points above.

Software and code

Policy information about availability of computer code

Data collection: No software used for data collection

Data analysis:
Assembly: Meraculous (v2.2.2.5) , PBJelly (v15.8.24), Meryl (v1.1), Merqury (v1.3)
Annotation: STAR (V2.5.2b, v2.7.8a), Stringtie (v1.3.3b),Trinity (v2.5.1) , GMAP (v2017-09-05) ,Exonerate (v2.2.0), Mikado (v1.2.1), Portcullis (v1.0.2), Trans-decoder (v5.5.0), RepeatModeller (2.0.1) , RepeatMasker (4.1.0) , PASA pipeline (v2.3.1), Eggnog-mapper (v2.1.5)
Gene family analyses: MMSeqs2 (r12-113e3), Broccoli (v1.1), MAFFT (v.7.471) , CLIPKIT (v1.1.6), IQTREE (v2.1.1), generax (v2.1.3), GenEra (v1.0.2),MMSeqs2 (r12-113e3)
Transcriptomics: featureCount from subreads package (v1.6.3),
HiC: bwa mem (v0.7.17),pairtools (v1.0.2) , juicer_tools (v1.22.01) , fanc python package (v0.9.23)
ATAC: bowtie2 (v.2.4.2), Generich (v0.6) HOMER (v5)
bulk RNAseq: BBTools (https://jgi.doe.gov/data-and-tools/software-tools/bbtools/bb-tools-user-guide/),
scRNA-seq: Cell Ranger (v7/0/1) , SAMAP (v1.0.2), Seurat (v5)
Methylome: BS-Seeker2 (v2.1.8) , Bowtie2 (v2.4.2), Sambamba (v1.0.1), CGmapTools (v0.1.3), DeepTools2 (v2)

For manuscripts utilizing custom algorithms or software that are central to the research but not yet described in published literature, software must be made available to editors and reviewers. We strongly encourage code deposition in a community repository (e.g. GitHub). See the Nature Portfolio guidelines for submitting code & software for further information.

Data

Policy information about availability of data

All manuscripts must include a data availability statement. This statement should provide the following information, where applicable:

- Accession codes, unique identifiers, or web links for publicly available datasets
- A description of any restrictions on data availability
- For clinical datasets or third party data, please ensure that the statement adheres to our policy

Supplementary files and codes used for data analysis has been deposited in zenodo: https://doi.org/10.5281/zenodo.13936459
Functional genomics data has been deposited in GEO GSE279488
The genome assembly and associated data have been deposited under project PRJNA1169515
All datasets are publicly availably.

Research involving human participants, their data, or biological material

Policy information about studies with human participants or human data. See also policy information about sex, gender (identity/presentation), and sexual orientation and race, ethnicity and racism.

Reporting on sex and gender	N/A
Reporting on race, ethnicity, or other socially relevant groupings	N/A
Population characteristics	N/A
Recruitment	N/A
Ethics oversight	N/A

Note that full information on the approval of the study protocol must also be provided in the manuscript.

Field-specific reporting

Please select the one below that is the best fit for your research. If you are not sure, read the appropriate sections before making your selection.

☒ Life sciences ☐ Behavioural & social sciences ☐ Ecological, evolutionary & environmental sciences

For a reference copy of the document with all sections, see nature.com/documents/nr-reporting-summary-flat.pdf

Life sciences study design

All studies must disclose on these points even when the disclosure is negative.

Sample size	Sample size is not relevant to the current study.
Data exclusions	No data exclusions.
Replication	For ISH, at least 3 individuals were used for qualitative assessment of reproducibility. For single cell and atac-seq data, several individuals were pooled for each stage.
Randomization	Randomisation was not applicable to the study.
Blinding	Blinding was not relevant to our study as comparison were performed by computer software not influenced by investigator.

Reporting for specific materials, systems and methods

We require information from authors about some types of materials, experimental systems and methods used in many studies. Here, indicate whether each material, system or method listed is relevant to your study. If you are not sure if a list item applies to your research, read the appropriate section before selecting a response.

Materials & experimental systems

n/a	Involved in the study
☒	☐ Antibodies
☒	☐ Eukaryotic cell lines
☒	☐ Palaeontology and archaeology
☐	☒ Animals and other organisms
☒	☐ Clinical data
☒	☐ Dual use research of concern
☒	☐ Plants

Methods

n/a	Involved in the study
☒	☐ ChIP-seq
☒	☐ Flow cytometry
☒	☐ MRI-based neuroimaging

Animals and other research organisms

Policy information about studies involving animals; ARRIVE guidelines recommended for reporting animal research, and Sex and Gender in Research

Laboratory animals	No laboratory animals used
Wild animals	Adult hermaphrodite Paraspadella gotoi were collected in Amakusa (Kyushu prefecture, Japan) and maintained in natural seawater. After spawning, juvenile chaetognaths were fed with increasingly older nauplii of the copepod Tigriopus californicus to match their size until capable of feeding on adults. All animals were sacrificed for the experiment of the paper (DNA-extraction, fixation, single cell sequencing).
Reporting on sex	Animals are hermaphrodites
Field-collected samples	We did not collect any field samples.
Ethics oversight	No ethics oversight needed

Note that full information on the approval of the study protocol must also be provided in the manuscript.

Plants

Seed stocks	*Report on the source of all seed stocks or other plant material used. If applicable, state the seed stock centre and catalogue number. If plant specimens were collected from the field, describe the collection location, date and sampling procedures.*
Novel plant genotypes	*Describe the methods by which all novel plant genotypes were produced. This includes those generated by transgenic approaches, gene editing, chemical/radiation-based mutagenesis and hybridization. For transgenic lines, describe the transformation method, the number of independent lines analyzed and the generation upon which experiments were performed. For gene-edited lines, describe the editor used, the endogenous sequence targeted for editing, the targeting guide RNA sequence (if applicable) and how the editor was applied.*
Authentication	*Describe any authentication procedures for each seed stock used or novel genotype generated. Describe any experiments used to assess the effect of a mutation and, where applicable, how potential secondary effects (e.g. second site T-DNA insertions, mosiacism, off-target gene editing) were examined.*
